# The impact of serum myokine profile on the outcome of peritoneal dialysis patients

**DOI:** 10.1186/s12882-026-05170-y

**Published:** 2026-07-02

**Authors:** Lixing Xu, Jack Kit-Chung Ng, Gordon Chun-Kau Chan, Winston Wing-Shing Fung, Wing-Fai Pang, Kai-Ming Chow, Cheuk-Chun Szeto

**Affiliations:** 1 Carol & Richard Yu Peritoneal Dialysis Research Centre, Department of Medicine & Therapeutics, Prince of Wales Hospital, Shatin, Hong Kong China; 2 Li Ka Shing Institute of Health Sciences (LiHS), Faculty of Medicine, The Chinese University of Hong Kong, Shatin, Hong Kong China; 3 Department of Medicine & Therapeutics, Prince of Wales Hospital, The Chinese University of Hong Kong, Shatin, NT, Hong Kong China

**Keywords:** Dialysis, End stage kidney failure, Chronic kidney disease, Frailty, Malnutrition, Sarcopenia, Inflammation

## Abstract

**Background:**

Peritoneal dialysis (PD) patients are at high risk for complications such as muscle loss and peritonitis. Individual myokines and functional assessment of PD patients are inconsistently reported to associate with poor prognosis, but their complex interactions for risk stratification have not been studied.

**Methods:**

In 194 new PD patients, we measured the serum levels of a myokine panel, metabolic and inflammatory markers, together with physical function scores. We employed k-cluster analysis on the result and analyzed patient survival, technique survival, peritonitis-free survival, hospitalisation rate and duration between the patient clusters.

**Results:**

There are two distinct patient clusters; cluster 1 showed a higher risk profile, with higher Morse Fall Scores, lower Norton scores, lower lean tissue mass, and different myokine levels. Cluster 2 is independently associated with better patient survival (adjusted hazard ratio 0.460, 95% confidence interval [CI] 0.237 to 0.894, *p* = 0.022) and peritonitis-free survival rate (AHR 0.417, 95% CI 0.205 to 0.846, *p* = 0.015). Cluster 2 also had lower hospitalisation rate (*p* = 0.022) and shorter duration of hospital stay (*p* = 0.014). Individual parameters used for the cluster analysis, however, were not independent predictors.

**Conclusion:**

A combined assessment of myokine profile and functional status of new PD patients is independently associated with patient survival, peritonitis-free survival, and hospitalization rates. Our results underscore the importance of a comprehensive, integrated approach to patient assessment, which can improve the risk stratification for this vulnerable population.

**Supplementary Information:**

The online version contains supplementary material available at https://doi.org/10.1186/s12882-026-05170-y.

## Introduction

Peritoneal dialysis (PD) is a life-saving renal replacement therapy for patients with end-stage kidney disease (ESKD). Compared to hemodialysis, PD offers greater flexibility and better preserves the patient’s residual renal function [1]. However, patients undergoing PD are at high risk for sarcopenia and peritonitis, which contribute to increased mortality and morbidity [2, 3]. The poor prognosis associated with PD is driven by a complex interplay of metabolic decline, chronic inflammation, and protein-energy wasting, making it challenging to identify specific risk factors [4, 5].

Several studies have highlighted the role of dysregulated myokines—such as Growth Differentiation Factor 15 (GDF-15), myostatin, follistatin, activin A, and irisin—in mediating chronic inflammation, muscle wasting, and metabolic dysfunction [6], and insulin resistance and systemic inflammation may further contribute to the risk [7–10]. Specifically, GDF-15 and serum C-reactive protein (CRP) are associated with reduced muscle performance and systemic inflammation [11]. Myostatin and activin A inhibit muscle growth upon binding to the Activin receptor II B (ActRIIB) [12, 13], and have an inverse correlation with muscle mass. Follistatin serves as a direct antagonist to myostatin [14]. Irisin is promyogenic and crucial for beige white adipose tissue [15, 16]. Myoglobin, on the other hands, is a marker of muscle breakdown [17].

Nonetheless, the prognostic relevance of these biomarkers remain uncertain. While myostatin was associated with sarcopenia and worse clinical outcomes [18], another study indicated that myostatin correlated negatively with mortality [19]. These inconsistencies may be caused by the inheriting drawbacks of traditional risk analysis, which often relies on univariable and multivariable Cox regression models, assuming linear relationships between each potential risk factor and survival outcomes. However, this approach may overlook the interrelationships and combined effects of these biomarkers. We hypothesize that there are complexed interactions among different biomarkers so that only a combination of dysregulated levels can better integrate their prognostic relevance. Thus, the objective of the current study is to systematically evaluate the effects of dysregulated myokines on the clinical outcome of new PD patients.

## Method

The study received approval from the Joint Chinese University of Hong Kong-New Territories East Cluster Clinical Research Ethics Committee (approval number CRE-2015.250). All procedures adhered to the guidelines outlined in the Declaration of Helsinki.

### Patient selection

This is a retrospective analysis of a prospective observational cohort study involving new adult PD patients from a single center. Patients were enrolled from 2020 to 2022. Patients were excluded if they had pacemakers, metallic prostheses, were scheduled for living donor kidney transplants, or planned to transfer to other medical centers within six months. After obtaining written informed consent, baseline assessments, including serum myokine profile, multi-frequency bioimpedance spectroscopy, peritoneal equilibration test, small solute clearance measurements, and other laboratory tests, were performed approximately four weeks after patients completed home PD training.

### Serum myokine profile

Blood samples were collected around the time of baseline assessments, which were prepared into serum samples and stored at -80 °C. Serum levels of a myokine panel were then measured in one batch using commercially available ELISA kits, following the manufacturers’ instructions. In this study, we assessed the serum concentrations of GDF-15, Activin A, irisin, myostatin, follistatin, myoglobin, and insulin. The specific ELISA kits employed are summarized in Table S1.

### Small solute clearance and other laboratory indices

Assessment of small solute clearance in dialysis was conducted through a 24-hour collection of dialysate and urine, as previously described [20]. The total weekly Kt/V was calculated using the standard formula. Residual glomerular filtration rate (GFR) was determined by averaging the 24-hour urinary urea and creatinine clearances [21]. Serum albumin levels were measured using the bromocresol purple method [22]. Additional laboratory tests, including plasma biochemistry and hemoglobin levels, were performed as part of routine clinical care.

### Multi-frequency bioimpedance spectroscopy

The bioimpedance spectroscopy (BIS) study was used to assess lean tissue mass (LTM) and adipose tissue mass (ATM) in peritoneal dialysis (PD) patients, as previously described [23]. Briefly, electrodes were placed on the patient’s right hand and foot while in a supine position. Measurements were conducted using the Body Composition Monitor (BCM, Fresenius Medical Care, Germany). Additionally, we assessed overhydration volume (OH), total body water (TBW), extracellular water (ECW), and intracellular water (ICW). The extracellular-to-intracellular (E/I) volume ratio was also calculated. All body composition measurements were performed during PD fluid dwell, as our prior study indicated that peritoneal dialysate had a negligible effect on the measurements [24].

### Follow-up and outcome parameters

The study cohort was followed till 1 July 2024. Throughout the follow-up, clinical management decisions were made independently by the treating clinicians without influence from the study. The primary outcomes included patient survival and technique survival. Secondary outcomes encompassed peritonitis-free survival, peritonitis rate, the number of hospital admissions, and total hospitalization days. In this study, technique failure was defined as a transition to hemodialysis, receiving a kidney transplant, or death. Transfers to other dialysis centers or recovery of renal function were considered censoring events. The peritonitis rate was calculated as the number of peritonitis episodes per patient-year [25].

### Statistical analysis

Statistical analyses were conducted utilizing SPSS version 28.0 (IBM Corporation, Armonk, NY) and GraphPad Prism (version 10.1.1, GraphPad Software, San Diego, CA). Data are presented as mean ± standard deviation or median (interquartile range), unless otherwise specified. To separate patients into clusters and subsequently analyse risk factors associated with the patients’ prognosis, we performed a K-means clustering method analysis of the patients’ biomarker and physical function profiles with R (version 2023.06.1 + 524), with Factoextra (version 1.0.7) and missForest packages (version 1.5). The silhouette index was used to determine the optimal number of clusters. Linear discriminant analysis (LDA) was then conducted to construct a scoring system that can quickly group patients into different clusters. The LDA score was calculated using variables that were previously chosen for constructing the cluster and also demonstrated significant differences between the two clusters. To describe the obtained clusters, we used the Mann-Whitney U-test to compare how the BIS measurements differ between the two groups. Spearman’s correlation coefficient was applied to examine the association between cluster number and other body composition metrics or biochemical parameters. The Kaplan-Meier method was employed to display data on patient survival, technique survival, and peritonitis-free survival rates. As described above, patients were stratified into two groups based on their cluster results, and their survival rates were compared using the log-rank test. Multivariate Cox regression models were further constructed to adjust for confounding clinical factors. In this analysis, we performed univariate Cox analysis for all available clinical and biochemical parameters; variables with p values < 0.1 in the univariate Cox regression were put into the multivariate Cox regression analysis, except those that were used for constructing the cluster to avoid collinearity issues. The peritonitis rate, the number of hospital admissions, and hospitalization duration between the two cluster groups were compared using the Mann-Whitney U-test. A p-value of less than 0.05 was considered statistically significant. All probabilities were two-tailed.

## Results

We studied 194 prevalent PD patients in the exploratory cohort. Their median duration of dialysis was 30 months (IQR 20 to 38 months). Myokine, profile of other biomarkers, and the result of physical assessment, were included for the K-means cluster analysis. The internal correlation matrix was summarized in Table S2. Two well-defined clusters of patients were identified based on the PCA (Figure S1A) and the Silhouette plot (Figure S1B), with 82 and 112 patients in cluster 1 and cluster 2, respectively. Their baseline demographic, clinical, and biochemical characteristics are summarized and compared in Tables 1 and 2, respectively. As compared to cluster 2, patients in cluster 1 had lower lean tissue mass, more fluid retention (overhydration volume and E/I ratio), and lower residual GFR.

**Table 1:** Baseline demographic and clinical characteristics

	Total	Cluster 1	Cluster 2	*P* values
No. of patients	194	82	112	
Sex (male: female)	116 to 78	42 to 40	74 to 38	0.037 ^b^
Age (years)	58.3 ± 11.3	67.3 ± 80.3	58.6 ± 11.8	< 0.0001^a^
Body built				
Weight (kg)	65.3 ± 14.1	61.3 ± 13.0	68.3 ± 14.3	0.0006 ^a^
Height (cm)	162.1 ± 9.0	159.4 ± 8.0	164.0 ± 9.2	< 0.0004 ^a^
Body mass index (kg/m^2^)	24.8 ± 4.9	24.0 ± 4.2	25.4 ± 5.3	0.051 ^a^
Blood pressure (mmHg)				
systolic	140.8 ± 19.3	142.4 ± 18.9	139.7 ± 19.6	0.348 ^a^
diastolic	76.2 ± 11.9	74.2 ± 11.2	77.6 ± 12.2	0.056 ^a^
Renal diagnosis, no. of case (%)				0.236 ^c^
diabetic nephropathy	93 (47.9%)	46 (56.1%)	47 (42.0%)	
glomerulonephritis	47 (24.2%)	16 (19.5%)	31 (27.7%)	
hypertension	28 (14.4%)	10 (12.2%)	18 (16.1%)	
urological problem	7 (3.6%)	2 (2.4%)	5 (4.5%)	
polycystic kidney disease	4 (2.1%)	0 (0%)	4 (3.6%)	
others or unknown	15 (7.7%)	8 (9.8%)	7 (6.3%)	
Major comorbidities, no. of case (%)				
diabetes mellitus	108 (55.7%)	54 (65.9%)	54 (48.2%)	0.015 ^c^
coronary artery disease	34 (17.5%)	17 (20.7%)	17 (15.2%)	0.315 ^c^
cerebrovascular accident	16 (8.2%)	9 (11.0%)	7 (6.3%)	0.237 ^c^
peripheral vascular disease	11 (5.7%)	6 (7.3%)	5 (4.5%)	0.396 ^c^
Charlson’s comorbidity index	5.9 ± 2.3	7 (5 to 8)	5 (4 to 7)	< 0.0001 ^a^
Morse Fall Scale	45 (35 to 62)	60 (55 to 75)	35 (15 to 40)	< 0.0001 ^b^
Norton Scale	19 (17 to 19)	17 (16 to 18)	19 (19 to 19)	< 0.0001 ^b^
Machine-assisted PD, no. of case (%)	49 (25.3%)	10 (12.2%)	39 (34.8%)	0.0003 ^c^

PD, peritoneal dialysis; GDP, glucose degradation products. Data was compared by ^a^Student’s t test, ^b^Mann-Whitney U test, and ^c^Chi-square test

**Table 2:** Baseline bioimpedance, biochemical, and nutritional information

	Total	Cluster 1	Cluster 2	*P* values
Myokine profile				
GDF-15 (ng/ml)	5.1 (3.4 to 8.0)	6.0 (3.6 to 8.9)	4.9 (2.9 to 6.8)	0.004 ^b^
Activin A (pg/ml)	193.5 (75.1 to 674.9)	172.4 (67.1 to 527.9)	211.8 (92.3 to 800.9)	0.230 ^b^
Irisin (pg/ml)	119.0 (56.3 to 222.3)	166.7 (73.6 to 350.6)	98.7 (38.0 to 171.7)	0.0005 ^b^
Myoglobin (ng/ml)	341.3 (252.2 to 488.1)	344.7 (236.6 to 499.2)	337.7 (254.6 to 485.4)	0.993 ^b^
Myostatin (ng/ml)	16.06 (12.02 to 25.70)	1.6 (0.9 to 2.2)	2.5 (1.7 to 3.8)	< 0.0001 ^b^
Follistatin (ng/ml)	1.4 (1.1 to 1.8)	1.6 (1.3 to 2.2)	1.2 (1.1 to 1.5)	< 0.0001 ^b^
Bioimpedance parameters				
lean tissue mass (kg)	41.1 ± 10.9	36.9 ± 9.4	44.0 ± 11.0	< 0.0001 ^a^
lean tissue percentage (%)	65.0 ± 13.9	62.3 ± 13.7	67.1 ± 13.7	0.016 ^a^
adipose tissue mass (kg)	18.7 (9.7 to 25.5)	18.9 (9.1 to 25.5)	17.7 (10.9 to 24.2)	0.909 ^b^
overhydration (L)	3.0 (1.6 to 5.0)	3.6 (2.2 to 5.6)	2.7 (1.4 to 4.3)	0.005 ^b^
E/I ratio	1.0 (0.9 to 1.1)	1.0 (0.9 to 1.1)	0.9 (0.8 to 1.0)	< 0.0001 ^b^
Fasting Plasma Glucose (mmol/L)	7.3 (5.8 to 11.2)	8.5 (6.4 to 12.6)	6.6 (5.3 to 10.0)	0.003 ^b^
Insulin (µU/ml)	4.1 (1.1 to 9.9)	3.7 (1.3 to 7.3)	4.7 (1.0 to 15.1)	0.335 ^b^
HOMA-IR	1.7 (0.3 to 4.9)	2.0 (0.4 to 3.7)	1.7 (0.3 to 5.0)	0.830 ^b^
C-reactive protein (mg/dL)	1.1 (0.5 to 2.6)	1.1 (0.5 to 2.9)	1.1 (0.5 to 2.4)	0.382 ^b^
Hemoglobin (g/dL)	10.6 ± 1.5	10.7 ± 1.5	10.4 ± 1.6	0.181 ^a^
Serum albumin (g/L)	28.5 ± 4.4	27.0 ± 4.5	29.6 ± 3.9	< 0.0001 ^a^
Total weekly Kt/V	2.1 (1.8 to 2.5)	2.2 (1.7 to 2.6)	2.1 (1.8 to 2.5)	0.339 ^b^
Residual GFR (ml/min/1.73m^2^)	4.5 (2.5 to 6.4)	3.7 (2.1 to 6.0)	4.9 (2.8 to 6.6)	0.030 ^b^
NPNA (g/kg/day)	1.1 (1.0 to 1.3)	1.1 (0.9 to 1.3)	1.1 (1.0 to 1.3)	0.370 ^b^
FEBM (kg)	21.0 (18.6 to 25.2)	20.3 (18.3 to 23.2)	21.4 (19.0 to 25.9)	0.127 ^b^
Peritoneal transport characteristics				
D/P4	0.6 ± 0.1	0.6 ± 0.1	0.6 ± 0.1	0.156 ^a^
MTAC creatinine (ml/min/1.73m2)	8.1 (6.1 to 11.9)	9.2 (6.7 to 12.6)	7.2 (5.2 to 10.7)	0.007 ^b^
Arterial pulse wave velocity (cm/sec)				
carotid-radial	10.8 ± 1.7	10.6 ± 1.6	10.9 ± 1.8	0.371 ^a^
carotid-femoral	11.0 ± 2.0	11.1 ± 2.0	10.8 ± 2.0	0.254 ^a^

D/P4, dialysate-to-plasma creatinine at 4 h; E/I ratio, extracellular to intracellular volume ratio; HOMA-IR, Homeostatic Model Assessment of Insulin Resistance Index; Kt/V, dialysis adequacy; GFR, glomerular filtration rate; MTAC, mass transfer area coefficient; NPNA, normalized protein nitrogen appearance; FEBM, fat-free-edema-free body mass; PWV, pulse-wave velocity. Data were presented as mean ± standard deviation or median (inter-quartile range), and compared by ^a^Student’s t-test, and ^b^Mann-Whitney U test

**Table 3:** Multi-variable Cox regression survival analysis patient survival

A. Patient survival						
	Univariate			Multivariate		
	unadjusted HR	95%CI	*P* values	adjusted HR	95%CI	*P* values
Patient Cluster (2 vs. 1)	0.343	0.195 to 0.605	0.0002	0.460	0.237 to 0.894	0.022
Sex (male to female)	1.257	0.719 to 2.198	0.422			
Body Weight	1.004	0.985 to 1.023	0.677			
Body Height	0.993	0.964 to 1.022	0.620			
Body Mass Index	1.018	0.975 to 1.064	0.414			
Waist circumference	1.024	0.995 to 1.054	0.104			
Hip circumference	1.023	0.984 to 1.062	0.251			
Systolic blood pressure	1.006	0.993 to 1.020	0.363			
Diastolic blood pressure	0.990	0.967 to 1.013	0.377			
Charlson’s comorbidity score	1.194	1.075 to 1.327	< 0.0001	1.144	1.012 to 1.293	0.031
Lean Tissue Mass	1.001	0.976 to 1.027	0.094	1.006	0.976 to 1.037	0.710
Adipose Tissue Mass	0.998	0.975 to 1.022	0.891			
Overhydration	1.182	1.098 to 1.274	< 0.0001	1.086	0.982 to 1.201	0.107
HOMA-IR	1.001	0.971 to 1.033	0.928			
Hemoglobin	0.866	0.722 to 1.039	0.123			
Serum albumin	0.886	0.836 to 0.939	< 0.0001	0.933	0.873 to 0.993	0.044
Total weekly Kt/V	1.027	0.771 to 1.369	0.854			
Residual GFR	0.930	0.834 to 1.041	0.210			
NPNA	0.814	0.329 to 2.013	0.657			
FEBM	0.999	0.980 to 1.019	0.921			
D/P4*	1.283	1.048 to 1.570	0.016	1.151	0.899 to 1.475	0.265
carotid-radial PWV	1.050	0.892 to 1.236	0.559			
carotid-femoral PWV	0.985	0.950 to 1.260	0.543			

B. Technique Survival						
	Univariate			Multivariate		
	unadjusted HR	95%CI	*P* values	adjusted HR	95%CI	*P* values
Patient Cluster (2 vs. 1)	0.517	0.322 to 0.830	0.006	0.735	0.437 to 1.237	0.247
Sex (male to female)	1.156	0.712 to 1.878	0.557			
Body Weight	1.005	0.989 to 1.022	0.540			
Body Height	0.996	0.971 to 1.022	0.773			
Body Mass Index	1.017	0.977 to 1.057	0.412			
Waist circumference	1.015	0.990 to 1.040	0.246			
Hip circumference	1.02	0.987 to 1.054	0.232			
Systolic blood pressure	1.006	0.994 to 1.018	0.356			
Diastolic blood pressure	1.003	0.983 to 1.023	0.803			
Charlson’s comorbidity score	1.158	1.054 to 1.271	0.002	1.131	1.018 to 1.256	0.022
Lean Tissue Mass	1.004	0.982 to 1.026	0.748			
Adipose Tissue Mass	1.001	0.981 to 1.022	0.919			
Overhydration	1.137	1.059 to 1.221	< 0.0001	1.067	0.977 to 1.166	0.150
HOMA-IR	1.002	0.975 to 1.030	0.284			
Hemoglobin	0.883	0.753 to 1.036	0.128			
Serum albumin	0.914	0.867 to 0.965	0.001	0.937	0.884 to 0.993	0.027
Total weekly Kt/V	0.983	0.747 to 1.294	0.904			
Residual GFR	0.945	0.859 to 1.040	0.224			
NPNA	0.652	0.278 to 1.531	0.326			
FEBM	1.001	0.985 to 1.071	0.903			
D/P4*	1.146	0.961 to 1.365	0.128			
carotid-radial PWV	0.986	0.850 to 1.143	0.851			
carotid-femoral PWV	0.971	0.885 to 1.144	0.411			

C. Peritonitis-free Survival						
	Univariate			Multivariate		
	unadjusted HR	95%CI	*P* values	adjusted HR	95%CI	*P* values
Patient Cluster (2 vs. 1)	0.559	0.351 to 0.888	0.014	0.417	0.205 to 0.846	0.015
Sex (male to female)	1.022	0.635 to 1.643	0.093	1.479	0.697 to 3.138	0.308
Body Weight	1.003	0.986 to 1.020	0.717			
Body Height	0.986	0.960 to 1.013	0.314			
Body Mass Index	1.030	0.981 to 1.081	0.241			
Waist circumference	1.025	0.999 to 1.052	0.055	1.020	0.988 to 1.052	0.221
Hip circumference	1.018	0.986 to 1.051	0.277			
Systolic blood pressure	0.994	0.982 to 1.006	0.325			
Diastolic blood pressure	0.993	0.973 to 1.013	0.509			
Charlson’s comorbidity score	1.103	0.996 to 1.219	0.060	1.035	0.879 to 1.218	0.680
Lean Tissue Mass	0.994	0.972 to 1.017	0.610			
Adipose Tissue Mass	1.005	0.984 to 1.026	0.645			
Overhydration	1.069	0.989 to 1.155	0.092	0.990	0.890 to 1.100	0.848
HOMA-IR	0.991	0.961 to 1.022	0.564			
Hemoglobin	0.818	0.702 to 0.952	0.009	0.873	0.701 to 1.087	0.225
Serum albumin	0.932	0.882 to 0.985	0.013	0.979	0.903 to 1.061	0.606
Total weekly Kt/V	1.005	0.757 to 1.335	0.973			
Residual GFR	0.953	0.872 to 1.041	0.287			
NPNA	1.256	0.621 to 2.542	0.525			
FEBM	1.000	0.984 to 1.015	0.952			
D/P4*	1.059	0.898 to 1.248	0.497			
carotid-radial PWV	0.989	0.857 to 1.141	0.881			
carotid-femoral PWV	1.002	0.933 to 1.205	0.874			

HR, hazard ratio; CI, confidence interval; GFR, glomerular filtration rate; NPNA, normalised protein nitrogen appearance; FEBM, fat-free edema-free mass; D/P4, dialysate-to-plasma creatinine concentration ratio at 4 hours; PWV, pulse-wave velocity. *Since the usual value of D/P4 ranges from 0.5 to 0.99, the result presented are hazard ratios for an increase in D/P4 by 0.1

NB. Factors that were used to define the patient clusters were used for model construction

## Patient and technique survival

**Fig. 1:**
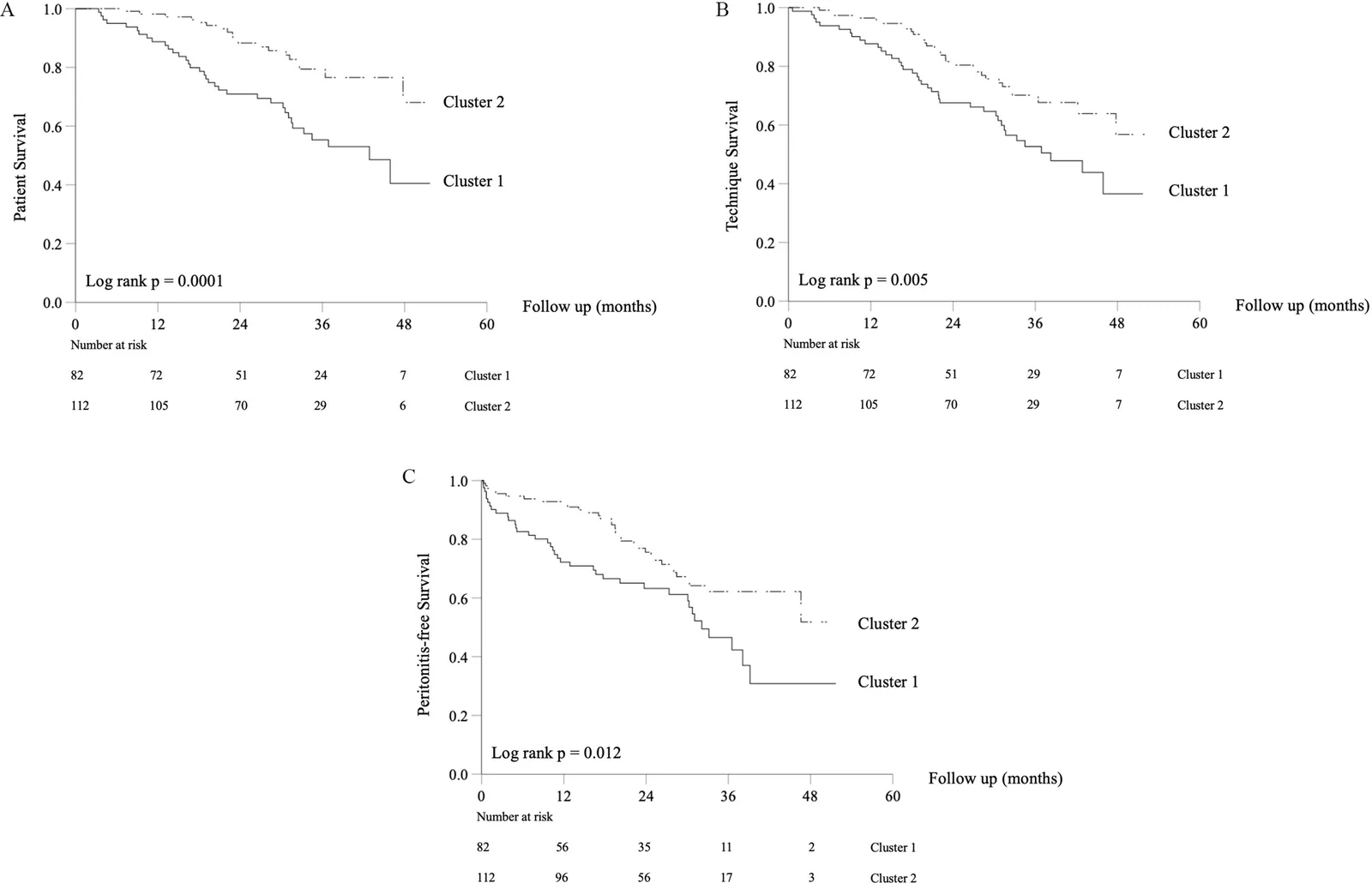
Kaplan–Meier survival curves comparing Cluster 1 versus Cluster 2: (**A**) patient survival over time, (**B**) technique survival over time, and (**C**) peritonitis-free survival over time. Each panel shows two stepwise survival trajectories with time on the x‑axis and survival probability on the y‑axis; differences between clusters are evaluated using the log-rank test

The median follow-up was 30.6 (IQR 20.2 to 37.5) months. During this period, 54 patients died. The causes of death were non-peritonitis infections (25 cases; including 13 cases of pneumonia, 8 cases of soft tissue infection, 2 cases of biliary infections, and 2 cases of septicemia of uncertain source), peritonitis (6 cases), ischemic heart disease (13 cases), cerebrovascular accident (4 cases), sudden cardiac death (1 case), termination of dialysis (3 cases), and other specific causes (2 cases). The result of the Cox regression survival analysis of the individual variable used for cluster construction is summarised in Table S3, but the factors are not included in the formal analysis due to collinearity. The two-year survival rates of cluster 1 and cluster 2 were 71.3% and 88.2%, respectively (log-rank test, *p* = 0.0001) (Fig. 1A). After adjusting for confounding clinical factors by multivariable Cox regression analysis, cluster 2 remained an independent predictor of patient survival (AHR = 0.471, 95% CI: 0.257 to 0.899, *p* = 0.022) (Table 3A).

During the follow-up period, another 16 patients were converted to haemodialysis, 11 received kidney transplants, 1 was transferred to other centers, and 1 had recovery of kidney function. The two-year technique survival rates of cluster 1 and cluster 2 were 68.0% and 80.2%, respectively (*p* = 0.005) (Fig. 1B). However, after adjusting for confounding clinical factors by multivariable Cox regression analysis, cluster 2 was not independently associated with technique survival (AHR = 0.735, 95% CI: 0.437 to 1.237, *p* = 0.247) (Table 3B).

### Peritonitis and hospitalisation

**Fig. 2:**
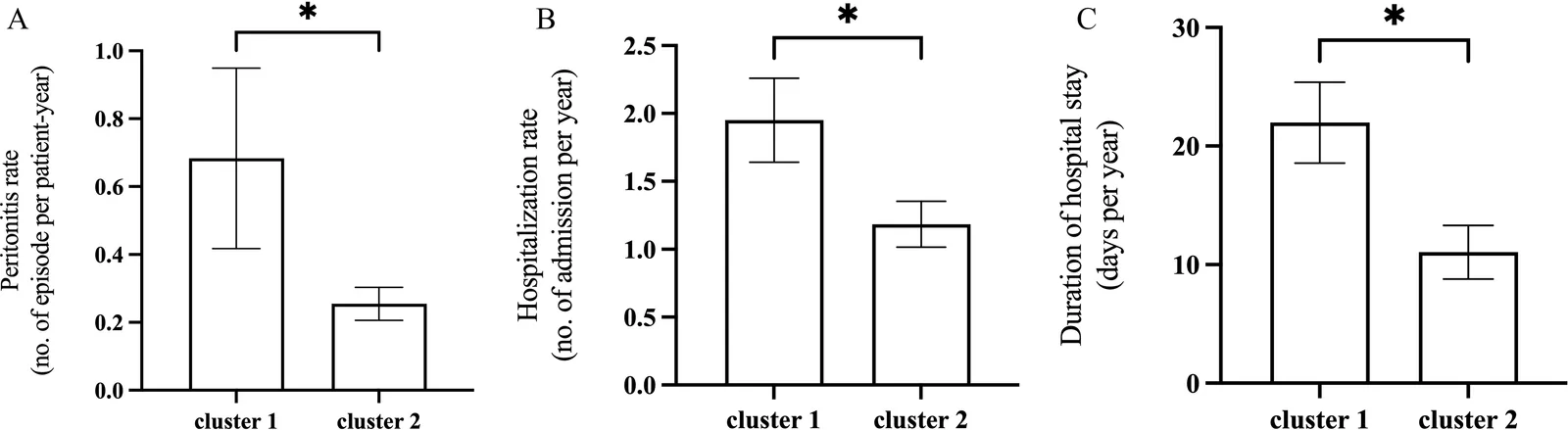
Comparison of the outcomes between Cluster 1 and Cluster 2 displayed in three panels: (**A**) peritonitis rate, (**B**) hospital admission rate, and (**C**) duration of hospital stay. Each panel includes error bars indicating the standard error of the mean. All data were adjusted for the duration of follow-up and analyzed using the Mann-Whitney test

During the follow-up period, there were 137 peritonitis episodes in 72 patients; 122 patients (62.9%) were peritonitis-free. The result of univariate and multivariate Cox regression analysis of peritonitis-free survival for individual variables used for cluster construction is summarised in Table S3C. The two-year peritonitis-free survival rates of clusters 1 and 2 were 63.8% and 75.3% (log-rank *p* = 0.012) (Fig. 1C). Similarly, cluster 2 showed a significantly lower peritonitis rate than cluster 1 (*p* = 0.026) (Fig. 2A). After adjusting for confounding factors, cluster 2 remained an independent predictor of a higher peritonitis-free survival rate than cluster 1 (AHR = 0.449, 95% CI 0.225 to 0.897, *p* = 0.023) (Table 3C).

During the follow-up period, there were 631 hospital admissions for a total of 5803 days. The overall peritonitis rate was 0.44 episodes per patient-year follow-up. Cluster 2 had significantly lower hospitalization rates than Cluster 1 (0.716 [IQR 0 to 1.429] vs. 1.267 [0.273 to 2.785] admission per year, *p* = 0.022), as well as shorter duration of hospitalization (3.335 [0 to 12.943] vs. 7.366 [0.705 to 31.670] days per year, *p* = 0.014) (Fig. 2B and C).

### Sensitivity analysis

We performed a sensitivity analysis by excluding 11 patients with BMI < 18. In this analysis, cluster 2 had higher patient survival, technique survival, and peritonitis-free survival rates than cluster 1 by univariate Cox regression (Table S4). With multi-variable Cox regression analysis to adjust for clinical confounding factors, cluster 2 remained independently associated with patient survival rates (AHR = 0.484, 95% CI 0.252 to 0.928, *p* = 0.029), but not technique survival or peritonitis-free survival rate. Another sensitivity assay was performed by including only the established prognostic factors, including Charlson’s comorbidity score, BMI, overhydration volume, serum albumin, and residual GFR, in the multivariate Cox regression analysis. In this analysis, cluster 2 remained an independent factor associated with patient survival (AHR = 0.450, 95% CI 0.237 to 0.855, *p* = 0.015), but not peritonitis-free survival (AHR = 0.661, 95% CI 0.397 to 1.101, *p* = 0.112) (Table S5).

### Scoring system development

We further developed a scoring system to simplify the procedure of cluster analysis so that it can be used and validated conveniently in other cohorts. We employed Linear Discriminant Analysis (LDA) to calculate the LDA score using age, myostatin, follistatin, irisin, GDF-15, FPG, Morse Fall Scale, and Norton Scale. These variables were chosen since they were previously used for constructing the cluster and also demonstrated significant differences between the two clusters. The final equation was:

$$\begin{aligned}\mathrm{LDA}\:\mathrm{score}&=0.053\:{}^*(\mathrm{GDF-15}\:[\mathrm{ng}/\mathrm{ml}])\cr&\quad+0.0017\:{}^*(\mathrm{Irisin}\:[\mathrm{ng}/\mathrm{ml}])\cr&\quad-0.26\:{}^*(\mathrm{Myostatin}\:[\mathrm{ng}/\mathrm{ml}])\cr&\quad+0.65\:{}^*(\mathrm{Follistatin}\:[\mathrm{ng}/\mathrm{ml}])\cr&\quad+0.0125\:{}^*(\mathrm{FPG}\:[\mathrm{ng}/\mathrm{ml}])\cr&\quad+0.027\:{}^*(\mathrm{Age}\:[\mathrm{years}])\cr&\quad+0.033\:{}^*(\mathrm{Morse}\:\mathrm{Fall}\:\mathrm{Scale})\cr&\quad-0.39\:{}^*(\mathrm{Norton}\:\mathrm{score})\cr&\quad+2.78\end{aligned}$$

With receiver operation characteristic (ROC) curve analysis, the area-under-curve (AUC) was 0.999, with a sensitivity of 100% and specificity of 98.2% at the cutoff 0.19. A score above the cutoff is equivalent to cluster 1, which is the high-risk group.

## Discussion

Although PD offers several advantages over hemodialysis (HD), it is often associated with complications such as sarcopenia and peritonitis, which increase both morbidity and mortality [26, 27]. Early identification of patients at higher risk for adverse events is therefore essential. While many studies have examined the prognostic value of individual biomarkers, the intricate interactions among these biomarkers in PD patients are often overlooked, potentially limiting their clinical relevance. In this study, we used cluster analysis to group PD patients according to their comprehensive biomarker profiles and evaluated how these distinct groups are associated with clinical outcomes.

With cluster analysis, we identified two distinct patient groups, with cluster 1 exhibiting a markedly worse risk profile. Specifically, patients in cluster 1 had significantly lower Norton Scale scores and myostatin levels, along with higher levels of GDF-15, irisin, follistatin, fasting plasma glucose, and Morse Fall Scale scores compared to those in cluster 2. Significant differences were also observed between the groups in demographic and biochemical characteristics. Notably, cluster 1 had lower muscle mass (as represented by LTM), increased fluid retention, and lower residual GFR. All of these factors probably contributed to poorer clinical outcomes [28–30].

We found that Cluster 1 had a significantly lower rates of patient and technique survival than Cluster 2, and remained an independent predictor of patient survival even after adjusting for potential confounders. Notably, none of the individual internal variables were independently associated with patient survival (Table S3A), while their combined profile serves as a strong predictor, suggesting that it is the synergistic interaction among myokine dysregulation, metabolic dysfunction, and functional impairment that drives adverse clinical outcomes, rather than the dysregulation of any single factor. This hypothesis may help explain the inconsistent findings regarding the clinical significance of many myokines, such as GDF-15 [11, 31, 32]. Although our study indicates that elevated GDF-15 levels alone have minimal effect on patient prognosis, when combined with other markers—such as lower myostatin and higher follistatin—there may be an increased risk of mortality.

After adjusting for potential confounding variables, cluster 1 remains an independent risk factor for peritonitis-free survival. Notably, none of the variables used to construct the clusters were individually associated with peritonitis-free survival (see Table S3C). Again, this finding highlights the fact that the level of individual myokine or clinical parameter is not sufficient for risk stratification because the interactions between myokines and clinical factors are crucial. Nonetheless, after adjustment for established risk factors, the association between the patient cluster and peritonitis-free survival was attenuated and became insignificant in the sensitivity analysis (*p* = 0.112 in Table S5C, as compared to *p* = 0.015 in Table 3C). In addition, cluster 2 had a significantly lower rate of hospitalization and shorter hospital stay than cluster 1. Given the differences in demographic and clinical parameters between the two clusters, it is also possible that cluster 1 represented a group of patients with general clinical frailty, making these patients more vulnerable to complications.

At first glance, it may seem paradoxical that cluster 1 has higher mortality and peritonitis rates and lower muscle mass, yet shows lower myostatin levels alongside higher follistatin and irisin levels compared to cluster 2. Myostatin is known to inhibit muscle growth [33], while follistatin and irisin are generally regarded as factors that promote muscle growth [34, 35]. However, recent studies reported that serum myostatin levels are actually positively correlated with muscle mass and survival [19, 36]. We believe this apparent contradiction arises because myostatin is primarily produced by muscle cells; in PD patients, higher myostatin levels may reflect greater muscle mass and better muscle quality. On the other hand, the increased levels of follistatin and irisin may result from upregulated synthesis in response to advanced oxidative stress and inflammation, which has been reported previously [37]. Therefore, elevated follistatin or irisin levels may indicate a failed compensatory response to preserve metabolic homeostasis and muscle function despite their reputation as “beneficial” myokines. Consequently, treatment with synthetic irisin or follistatin, though promising in animal studies [38], may not be effective in this context.

This study has several limitations. First, it is a single-center retrospective analysis, and there is a need to further validate cluster analysis methods in larger, more diverse cohorts. Furthermore, we only assessed the baseline measurements and did not account for longitudinal changes in the myokine profiles over time. While we demonstrated significant differences in internal variables between the two clusters, we did not identify specific cutoff values for each factor to aid in cluster identification. It remains challenging to identify at-risk populations outside our cohort. Although we used LDA analysis to construct a scoring system with high specificity and sensitivity, the equation has not yet been validated externally. In this study, non-peritonitis infection was a major cause of death, which was consistent with the registry report in our locality [39] but may be considerably different in other parts of the world. It remains unknown how well the model can predict the patient’s clinical outcomes outside our cohort. Future research should therefore use multiple cohorts with larger sample sizes to validate our findings and establish clinically relevant cutoff values.

In conclusion, our findings indicate that a combined assessment of myokine profile and functional status of new PD patients is independently associated with patient survival, peritonitis-free survival, and hospitalization rates. Our results underscore the importance of a comprehensive, integrated approach to patient assessment, which can improve the risk stratification for this vulnerable population.

## Supplementary Information

Below is the link to the electronic supplementary material.

Supplementary Material 1

Supplementary Material 2

Supplementary Material 3

## Data Availability

The data underlying this article are publicly available at the following site: https://github.com/szetocc/myokine_cluster/blob/main/Myokine%20data.xlsx.

## Declarations

### Abbreviations

Please refer to descriptions in the main text.
