# Supplementary material for: The impact of serum myokine profile on the outcome of peritoneal dialysis patients
Source: BMC Nephrol. 2026 Jul 2;27:560. doi: 10.1186/s12882-026-05170-y (PMC13602474; doi:10.1186/s12882-026-05170-y)
Supplement: Supplementary file 1 [file 12882_2026_5170_MOESM3_ESM.docx]

# Table S1. Basic information about the ELISA kits used to quantify biomarkers

| Biomarker | Producer | Catalog Number | Detection Range | Coefficient of Variation (%) |
| --- | --- | --- | --- | --- |
| GDF-15 | Life Technologies Limited | EHGDF15 | 1.1-800 pg/ml | < 10% |
| Activin A | Life Technologies Limited | EHACTIVINA | 20.48-5000 pg/ml | < 10% |
| Irisin | NOVUS | NBP3-08117 | 0.16-10 ng/ml | < 10% |
| Myostatin | R&D Systems Inc | DGDF80 | 31.3-2000 pg/ml | < 10% |
| Follistatin | R&D Systems Inc | DFN00 | 250-16000 pg/ml | < 10% |
| Myoglobin | Life Technologies Limited | EEL001 | 0.31-20 ng/ml | < 10% |
| Insulin | ImmunoDiagnostic Limited | 31380 | 3-120 µU/mL | < 10% |

GDF-15, Growth Differentiation Factor 15.

# Table S2. Internal correlation matrix of factors used for cluster analysis

|  | Activin A | Irisin | Myoglobin | Myostatin | Follistatin | FPG | Insulin | CRP | Age | Temperature | Morse | Norton | SaO2 |
| --- | --- | --- | --- | --- | --- | --- | --- | --- | --- | --- | --- | --- | --- |
| GDF-15 | r = 0.027  p = 0.710 | r = 0.137  p = 0.056 | r = 0.204  p = 0.004 | r = -0.119  p = 0.100 | r = 0.154  p = 0.032 | r = 0.082  p = 0.256 | r = 0.004  p = 0.956 | r = 0.148  p = 0.049 | r = 0.094  p = 0.191 | r = -0.011  p = 0.877 | r = 0.135  p = 0.064 | r = -0.057  p = 0.438 | r = 0.060  p = 0.417 |
| Activin A |  | r = 0.003  p = 0.650 | r = -0.043  p = 0.555 | r = 0.011  p = 0.878 | r = -0.047  p = 0.516 | r = -0.015  p = 0.832 | r = 0.107  p = 0.155 | r = -0.038  p = 0.615 | r = -0.061  p = 0.401 | r = -0.158  p = 0.030 | r = -0.119  p = 0.103 | r = -0.051  p = 0.488 | r = 0.046  p = 0.535 |
| Irisin |  |  | r = 0.060  p = 0.402 | r = -0.045  p = 0.533 | r = 0.126  p = 0.081 | r = 0.068  p = 0.349 | r = -0.003  p = 0.965 | r = 0.082  p = 0.279 | r = 0.034  p = 0.635 | r = 0.055  p = 0.450 | r = 0.095  p = 0.195 | r = -0.031  p = 0.674 | r = 0.029  p = 0.692 |
| Myoglobin |  |  |  | r = 0.182  p = 0.011 | r = 0.136  p = 0.058 | r = 0.072  p = 0.318 | r = 0.080  p = 0.287 | r = 0.099  p = 0.190 | r = -0.072  p = 0.320 | r = -0.006  p = 0.928 | r = 0.019  p = 0.792 | r = -0.021  p = 0.771 | r = 0.032  p = 0.662 |
| Myostatin |  |  |  |  | r = -0.250  p = 0.0004 | r = 0.028  p = 0.697 | r = 0.039  p = 0.608 | r = -0.008  p = 0.911 | r = -0.083  p = 0.248 | r = 0.023  p = 0.757 | r = -0.211  p = 0.004 | r = 0.179  p = 0.014 | r = 0.059  p = 0.426 |
| Follistatin |  |  |  |  |  | r = 0.051  p = 0.479 | r = -0.127  p = 0.092 | r = 0.078  p = 0.303 | r = 0.066  p = 0.359 | r = 0.011  p = 0.880 | r = 0.104  p = 0.155 | r = -0.190  p = 0.009 | r = 0.046  p = 0.539 |
| FPG |  |  |  |  |  |  | r = 0.306  p<0.0001 | r = 0.126  p = 0.096 | r = 0.205  p = 0.004 | r = -0.012  p = 0.866 | r = 0.167  p = 0.022 | r = -0.205  p = 0.005 | r = -0.096  p = 0.199 |
| Insulin |  |  |  |  |  |  |  | r = 0.078  p = 0.322 | r = -0.007  p = 0.923 | r = 0.107  p = 0.158 | r = 0.022  p = 0.775 | r = 0.120  p = 0.116 | r = 0.021  p = .788 |
| CRP |  |  |  |  |  |  |  |  | r = 0.193  p = 0.010 | r = -0.023  p = 0.758 | r = -0.004  p = 0.957 | r = -0.022  p = 0.778 | r = -0.033  p = 0.670 |
| Age |  |  |  |  |  |  |  |  |  | r = -0.062  p = 0.395 | r = 0.356  p<0.0001 | r = -0.367  p<0.0001 | r = -0.112  p = 0.132 |
| Temperature |  |  |  |  |  |  |  |  |  |  | r = -0.005  p = 0.951 | r = 0.024  p = 0.749 | r = 0.046  p = 0.536 |
| Morse |  |  |  |  |  |  |  |  |  |  |  | r = -0.578  p<0.0001 | r = -0.090  p = 0.228 |
| Norton |  |  |  |  |  |  |  |  |  |  |  |  | r = 0.239  p = 0.001 |

GDF-15, growth differentiation factor 15; FPG, fasting plasma glucose level; CRP, C-reactive protein, Morse, Morse Fall Scale; Norton, Norton Scale; SaO2, peripheral oxygen levels.

# Table S3. Cox regression analysis for parameters used for cluster analysis

## A. Patient Survival

|  | Unadjusted HR | P values | Adjusted HR | 95% CI | P values |
| --- | --- | --- | --- | --- | --- |
| Age | 1.024 | 0.078 | 0.997 | 0.960 to 1.036 | 0.877 |
| Morse Fall Scale | 1.018 | 0.006 | 0.999 | 0.983 to 1.016 | 0.945 |
| Norton Scale | 0.739 | < 0.0001 | 0.898 | 0.745 to 1.084 | 0.263 |
| Temperature | 1.095 | 0.693 |  |  |  |
| Peripheral Oxygen | 1.034 | 0.399 |  |  |  |
| GDF-15 * | 1.047 | 0.839 |  |  |  |
| Activin A * | 0.916 | 0.130 |  |  |  |
| Irisin * | 1.248 | 0.112 |  |  |  |
| Myoglobin * | 1.419 | 0.181 |  |  |  |
| Myostatin* | 0.527 | 0.017 | 0.704 | 0.367 to 1.351 | 0.291 |
| Follistatin * | 2.497 | 0.011 | 0.903 | 0.368 to 2.214 | 0.824 |
| Fasting Plasma Glucose | 1.034 | 0.045 | 1.018 | 0.970 to 1.069 | 0.469 |
| Insulin | 0.999 | 0.909 |  |  |  |
| C-reactive protein | 1.028 | 0.474 |  |  |  |

## B. Technique Survival

|  | Unadjusted HR | P values | Adjusted HR | 95% CI | P values |
| --- | --- | --- | --- | --- | --- |
| Age | 1.011 | 0.316 |  |  |  |
| Morse Fall Scale | 1.012 | 0.028 | 0.999 | 0.986 to 1.013 | 0.887 |
| Norton Scale | 0.798 | 0.001 | 0.918 | 0.769 to 1.095 | 0.340 |
| Temperature | 1.274 | 0.217 |  |  |  |
| Peripheral Oxygen | 1.038 | 0.351 |  |  |  |
| GDF-15 * | 1.282 | 0.200 |  |  |  |
| Activin A * | 0.936 | 0.207 |  |  |  |
| Irisin * | 1.223 | 0.096 | 1.083 | 0.851 to 1.378 | 0.516 |
| Myoglobin * | 1.185 | 0.468 |  |  |  |
| Myostatin* | 2.063 | 0.024 | 0.896 | 0.544 to 1.479 | 0.667 |
| Follistatin * | 0.66 | 0.078 | 1.005 | 0.464 to 2.176 | 0.990 |
| Fasting Plasma Glucose | 1.025 | 0.120 |  |  |  |
| Insulin | 1.000 | 0.965 |  |  |  |
| C-reactive protein | 1.014 | 0.703 |  |  |  |

## C. Peritonitis-free Survival

|  | Unadjusted HR | P values | Adjusted HR | 95% CI | P values |
| --- | --- | --- | --- | --- | --- |
| Age | 1.002 | 0.876 |  |  |  |
| Morse Fall Scale | 1.018 | 0.125 |  |  |  |
| Norton Scale | 0.927 | 0.339 |  |  |  |
| Temperature | 1.021 | 0.918 |  |  |  |
| Peripheral Oxygen | 1.008 | 0.549 |  |  |  |
| GDF-15 * | 1.022 | 0.913 |  |  |  |
| Activin A * | 0.910 | 0.063 | 0.884 | 0.732 to 1.068 | 0.202 |
| Irisin * | 1.095 | 0.439 |  |  |  |
| Myoglobin * | 1.137 | 0.578 |  |  |  |
| Myostatin* | 1.656 | 0.115 |  |  |  |
| Follistatin * | 0.655 | 0.060 | 1.169 | 0.459 to 2.979 | 0.743 |
| Fasting Plasma Glucose | 1.020 | 0.240 |  |  |  |
| Insulin | 0.994 | 0.590 |  |  |  |
| C-reactive protein | 1.013 | 0.708 |  |  |  |

GDF-15, growth differentiation factor 15; HR, hazard ratio; CI, confidence interval.

In the computation of the adjusted hazard ratio, Charlson’s comorbidity score, lean tissue mass, overhydration, albumin, and D/P4 levels were also included in the model. *data were log-transformed for analysis due to severe skewness.

# Table S4. Sensitivity analysis with multi-variable Cox regression survival analysis by excluding patients with BMI ≤ 18

## A. Patient survival

|  | Univariate |  | Multivariate |  |  |
| --- | --- | --- | --- | --- | --- |
|  | unadjusted HR | P values | adjusted HR | 95%CI | P values |
| Patient Cluster (2 vs 1) | 0.353 | 0.0004 | 0.471 | 0.246 to 0.903 | 0.023 |
| Sex (male to female) | 1.215 | 0.512 |  |  |  |
| Body Weight | 1.004 | 0.700 |  |  |  |
| Body Height | 0.988 | 0.427 |  |  |  |
| Body Mass Index | 1.021 | 0.373 |  |  |  |
| Hip circumference | 1.022 | 0.343 |  |  |  |
| Systolic blood pressure | 1.008 | 0.289 |  |  |  |
| Diastolic blood pressure | 0.992 | 0.541 |  |  |  |
| Charlson’s comorbidity score | 1.197 | 0.001 | 1.146 | 1.008 to 1.302 | 0.037 |
| Lean Tissue Mass | 1.003 | 0.839 |  |  |  |
| Adipose Tissue Mass | 0.995 | 0.702 |  |  |  |
| Overhydration | 1.185 | 0.0001 | 1.111 | 1.012 to 1.220 | 0.027 |
| HOMA-IR | 1.001 | 0.962 |  |  |  |
| Hemoglobin | 0.870 | 0.140 |  |  |  |
| Serum albumin | 0.893 | 0.0003 | 0.944 | 0.879 to 1.015 | 0.118 |
| Total weekly Kt/V | 1.109 | 0.443 |  |  |  |
| Residual GFR | 0.936 | 0.249 |  |  |  |
| NPNA | 0.900 | 0.822 |  |  |  |
| FEBM | 0.998 | 0.826 |  |  |  |
| D/P4* | 1.317 | 0.012 | 1.186 | 0.913 to 1.540 | 0.201 |
| Carotid-Radius PWV | 1.032 | 0.715 |  |  |  |
| Carotid-Femur PWV | 1.071 | 0.361 |  |  |  |

## B. Technique survival

|  | Univariate |  | Multivariate |  |  |
| --- | --- | --- | --- | --- | --- |
|  | unadjusted HR | P values | adjusted HR | 95%CI | P values |
| Patient Cluster (2 vs 1) | 0.540 | 0.013 | 0.744 | 0.433 to 1.278 | 0.285 |
| Sex (male to female) | 1.139 | 0.615 |  |  |  |
| Body Weight | 1.006 | 0.501 |  |  |  |
| Body Height | 0.993 | 0.599 |  |  |  |
| Body Mass Index | 1.020 | 0.345 |  |  |  |
| Systolic blood pressure | 1.008 | 0.233 |  |  |  |
| Diastolic blood pressure | 1.008 | 0.468 |  |  |  |
| Charlson’s comorbidity score | 1.161 | 0.003 | 1.128 | 1.010 to 1.259 | 0.033 |
| Lean Tissue Mass | 1.006 | 0.597 |  |  |  |
| Adipose Tissue Mass | 0.999 | 0.901 |  |  |  |
| Overhydration | 1.141 | 0.0004 | 1.074 | 0.983 to 1.174 | 0.114 |
| HOMA-IR | 1.002 | 0.895 |  |  |  |
| Hemoglobin | 0.887 | 0.143 |  |  |  |
| Serum albumin | 0.922 | 0.004 | 0.950 | 0.890 to 1.014 | 0.121 |
| Total weekly Kt/V | 1.058 | 0.667 |  |  |  |
| Residual GFR | 0.945 | 0.256 |  |  |  |
| NPNA | 0.900 | 0.822 |  |  |  |
| FEBM | 0.998 | 0.826 |  |  |  |
| D/P4* | 1.317 | 0.012 | 1.048 | 0.847 to 1.296 | 0.668 |
| Carotid-Radius PWV | 1.032 | 0.715 |  |  |  |
| Carotid-Femur PWV | 1.071 | 0.361 |  |  |  |

## C. Peritonitis-free survival

|  | Univariate |  | Multivariate |  |  |
| --- | --- | --- | --- | --- | --- |
|  | unadjusted HR | P values | adjusted HR | 95%CI | P values |
| Patient Cluster (2 vs 1) | 0.553 | 0.015 | 0.741 | 0.432 to 1.270 | 0.275 |
| Sex (male to female) | 1.034 | 0.897 |  |  |  |
| Body Weight | 1.006 | 0.528 |  |  |  |
| Body Height | 0.986 | 0.312 |  |  |  |
| Body Mass Index | 1.042 | 0.119 |  |  |  |
| Systolic blood pressure | 0.993 | 0.285 |  |  |  |
| Diastolic blood pressure | 0.990 | 0.355 |  |  |  |
| Charlson’s comorbidity score | 1.111 | 0.049 | 1.138 | 1.019 to 1.271 | 0.022 |
| Lean Tissue Mass | 0.996 | 0.728 |  |  |  |
| Adipose Tissue Mass | 1.007 | 0.542 |  |  |  |
| Overhydration | 1.065 | 0.127 |  |  |  |
| HOMA-IR | 0.992 | 0.604 |  |  |  |
| Hemoglobin | 0.834 | 0.021 | 0.893 | 0.757 to 1.054 | 0.180 |
| Serum albumin | 0.947 | 0.061 | 0.940 | 0.886 to 0.996 | 0.037 |
| Total weekly Kt/V | 1.123 | 0.423 |  |  |  |
| Residual GFR | 0.945 | 0.256 |  |  |  |
| NPNA | 1.553 | 0.205 |  |  |  |
| FEBM | 0.998 | 0.821 |  |  |  |
| D/P4* | 1.054 | 0.549 |  |  |  |
| Carotid-Radius PWV | 0.996 | 0.960 |  |  |  |
| Carotid-Femur PWV | 1.050 | 0.476 |  |  |  |

HR, hazard ratio; CI, confidence interval; GFR, glomerular filtration rate; NPNA, normalised protein nitrogen appearance; FEBM, fat-free edema-free mass; D/P4, dialysate-to-plasma creatinine concentration ratio at 4 hours; PWV, pulse-wave velocity. *For every 0.1 unit increase in D/P4.

NB. Factors that were used to define the patient clusters were used for model construction.

# Table S5. Sensitivity analysis with multi-variable Cox regression survival analysis with established prognostic factors

## A. Patient survival

|  | adjusted HR | 95%CI | P values |
| --- | --- | --- | --- |
| Patient Cluster (2 vs 1) | 0.450 | 0.237 to 0.855 | 0.015 |
| Charlson’s comorbidity score | 1.133 | 0.999 to 1.284 | 0.051 |
| Body Mass Index | 1.019 | 0.966 to 1.076 | 0.488 |
| Overhydration | 1.103 | 1.000 to 1.217 | 0.050 |
| Serum albumin | 0.908 | 0.848 to 0.973 | 0.006 |
| Residual GFR | 1.048 | 0.928 to 1.183 | 0.450 |

## B. Technique survival

|  | adjusted HR | 95%CI | P values |
| --- | --- | --- | --- |
| Patient Cluster (2 vs 1) | 0.706 | 0.414 to 1.205 | 0.202 |
| Charlson’s comorbidity score | 1.122 | 1.007 to 1.250 | 0.037 |
| Body Mass Index | 1.017 | 0.973 to 1.063 | 0.462 |
| Overhydration | 1.064 | 0.970 t0 1.167 | 0.190 |
| Serum albumin | 0.933 | 0.877 to 0.992 | 0.027 |
| Residual GFR | 1.011 | 0.912 to 1.121 | 0.835 |

## C. Peritonitis-free survival

|  | adjusted HR | 95%CI | P values |
| --- | --- | --- | --- |
| Patient Cluster (2 vs 1) | 0.661 | 0.397 to 1.101 | 0.112 |
| Charlson’s comorbidity score | 1.042 | 0.927 to 1.172 | 0.486 |
| Body Mass Index | 1.046 | 0.991 to 1.104 | 0.103 |
| Overhydration | 0.985 | 0.898 to 1.080 | 0.748 |
| Serum albumin | 0.938 | 0.880 to 1.000 | 0.051 |
| Residual GFR | 0.982 | 0.893 to 1.079 | 0.701 |
